# Prevention of Preterm Labour: 2011 Update on Tocolysis

**DOI:** 10.1155/2011/941057

**Published:** 2011-11-15

**Authors:** C. Hubinont, F. Debieve

**Affiliations:** Department of Obstetrics, Saint-Luc University Hospital, 10 Avenue Hippocrate, 1200 Bruxelles, Belgium

## Abstract

The aim of this paper is to review available data about drugs for preventing preterm labour. Tocolytic therapy includes **β** adrenergic receptor agonists, NO donors, magnesium sulphate, prostaglandin-synthase inhibitors, oxytocin receptor antagonists, calcium-channel blockers, progesterone, 17-**α**-hydroxyprogesterone caproate, and antibiotics. Their specific effects on myometrial contractility, their safety, their efficiency, and side effects profile for the mother and the fetus are presented. The main question of why and for what reasons tocolysis should be administrated is discussed.

## 1. Introduction

Preterm delivery is defined by a birth occurring before 37 weeks of gestation or before 259 days from the last menstrual period. Prematurity is multifactorial and its incidence has increased during the last decade in most occidental countries, probably due to increased risk factors responsible for elective prematurity [1–3]. 

The mechanisms for preterm labour are still unclear. It could be associated either with a premature activation of the physiological contracting process or with a pathological factor responsible for uterine contractions, leading to preterm delivery [1–3].

Among identified pathways for preterm labour, there are uterine overdistension due to multiple pregnancies or polyhydramnios, placental ischaemia, cervical disease, immunologic and allergic phenomena, decidual or retroplacental haemorrhage, fetal endocrine activation intrauterine infections, and inflammatory processes. Elective prematurity due to maternal or foetal conditions is becoming a significant cause [1–4].

Tocolytic drugs have been available for several decades but their actions are directed toward the effects and not the causes of preterm labour [1, 3, 5].

Therapeutic strategies available in the literature for stopping preterm labour are discussed in this paper. Their efficacy depend on an early and accurate diagnosis of the condition, the fetal fibronectin, and cervical length ultrasonography [2].

Drugs safety and side effect profile is a major concern not only for the pregnant women but also for the foetus [4–6]. In some clinical conditions such as abruptio and chorioamnionitis, inhibition of uterine contractions and birth delay may be more harmful in terms of outcome and should be avoided [2, 3]. Another concern is the administration route and the optimal range of gestational age for these treatments [5]. 

Tocolysis aims not only to inhibit uterine contractions but also to allow a safe transfer of the pregnant patient to a tertiary care centre. It gives the opportunity to administrate corticosteroids for preventing neonatal risks associated with prematurity [5–7]. 

## 2. Mechanisms of Tocolysis 

Myometrial contractility is a complex process based on myocytes function. It involves the presence of hormonal receptors, ions channels, intercell gap junctions, and regulatory proteins such as oxytocin, endothelin, tachykinin, and angiotensin [8, 9]. The increase of intracellular calcium concentration is essential for the uterine smooth muscle contraction [9]. 

**Figure 1:**
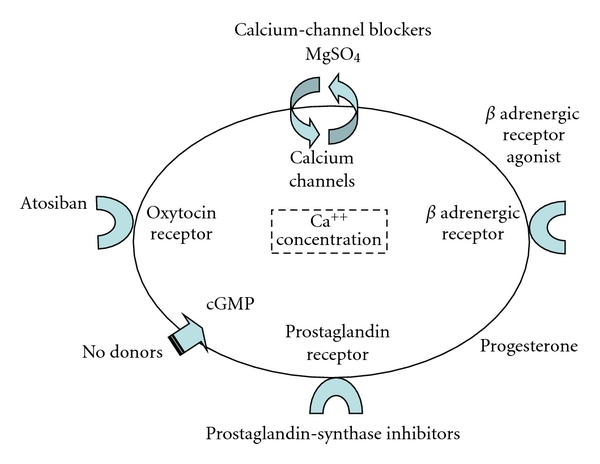
Mechanisms of action for tocolytics.

As shown on Figure 1, uterine relaxation may be obtained by interfering with an intracellular messenger responsible for contractile proteins effects: *β* adrenergic receptor agonists, nitric oxide (NO) donors, magnesium sulphate and calcium channel blockers are tocolytic drugs aiming to this [1, 2, 6, 9]. Another pathway involves the inhibition of contracting factors synthesis or effect. Atosiban, an oxytocin receptor antagonist and prostaglandin-synthetase inhibitors have this effect by interfering with endogenous myometrial stimulators [1, 2, 6, 9].

## 3. Types of Tocolytic Treatment

### 3.1. *β* Adrenergic Receptor Agonists

Selective *β* 2 agonists such as ritodrine and salbutamol have been used in clinical practice for preterm labour since the 1980s. These drugs impair intracellular cyclic AMP concentration and facilitate myometrial relaxation [9, 10]. Randomized controlled studies and meta-analysis reported that these agents were more efficient than placebo for delaying preterm birth for two days. Unfortunately, no benefit for long-term (tocolytic effect restricted to 7 days) and perinatal mortality and morbidity rate was found [5, 10, 11]. Moreover, even with selective *β* 2 adrenergic receptor agonists, there are significant maternal side effects reported such as tachycardia, dyspnoea, hypokalemia, hyperglycemia, and chest pain [5, 6, 9–12]. In conclusion, despite their efficiency, *β* 2 agonists' safety profile is a real concern responsible for therapy discontinuation and choosing alternative tocolytic drugs.

### 3.2. NO Donors

NO is a powerful vasodilator synthesized during an amino acid oxidation process catalysed by NO synthase. It is present in myometrial cells and increases cGMP content by interaction with guanylyl cyclase. There is a specific link between NO production and uterine relaxation [8, 9].

Transdermal nitroglycerin administration has been used in preterm labour but only in small series. It was associated to a better tocolytic effect than placebo on delaying delivery for two days. Its effect was similar to ritodrine [2–5]. As there is no large randomized studies available, NO is not used in clinical routine.

### 3.3. Magnesium Sulphate

The relaxant effect of Magnesium sulphate in vitro and in vivo on human uterine contractility has been widely reported. As magnesium is a calcium antagonist, it decreases calcium intracellular concentration and inhibits contraction process [2, 4, 9]. However, in 2002, a meta-analysis based on 881 patients did not evidence any benefit of Magnesium sulphate administration over placebo use in preterm labour [13]. As the drug is crossing the placenta, there were concerns about fetal safety. An increased risk of perinatal death and neonatal adverse effects including neurological and metabolic disorders were reported in some trials using Magnesium sulphate treatment at high dosage [6, 13]. It can also affect maternal neuromuscular system. Over a serum concentration of 9 mg/dL, there is a high toxicity risk resulting in respiratory depression and disappearance of reflexes. There is no evidence any more to recommend this drug as a first-line tocolytic agent [2, 6, 13, 14]. 

However, when administered prophylactically at low dose, it was reported to have a neonatal neuroprotective effect in a randomized multicentre trial [15] but this effect should be confirmed in the next future on large randomised controlled studies [16].

### 3.4. Prostaglandin-Synthase Inhibitors

Prostaglandin-synthase or cyclooxygenase (COX) isoforms COX-1 and -2 are essential enzymes for converting arachidonic acid to prostaglandins. Prostaglandins are well-known uterine contraction inducer by enhancing myometrial gap junction and increasing intracellular calcium concentration [2, 4, 5, 9]. Indomethacin, a nonspecific COX inhibitor, has been reported in studies and in a recent meta-analysis to be an efficient tocolytic drug compared to placebo, significantly delaying preterm delivery [11]. It can be administrated rectally or orally. Its use should be restricted in duration and limited to pregnancies below 32 weeks because of fetal ductus arteriosus closure risk and decreased urine production responsible for oligohydramnios [3, 5, 6, 17]. These treatments also have maternal side effects including gastric ulcer or asthma recurrence [3, 5, 6]. COX-2 inhibitors such as nimesulide or rofecoxib have been studied in animal but not yet in humans and are not actually recommended for preventing preterm labour in clinical practice [18]. In conclusion, indomethacin is an efficient tocolytic drug with no serious adverse drug reaction and is indicated for short-term effect during the second trimester of pregnancy.

### 3.5. Oxytocin Receptor Antagonists

These agents are in competition with the myometrial and decidual oxytocin receptors. The only drug used in clinical practice is atosiban. It blocks in a reversive manner the intracytoplasmic calcium release associated with contractions and downregulates prostaglandin synthesis [2, 9]. A first multicentric randomised trial comparing atosiban and ritodrine demonstrated a similar tocolytic effect but fewer adverse effects with atosiban [4, 6]. A meta-analysis published in 2005 reported no benefit in terms of preterm delivery rate and neonatal outcome in 1695 patients treated either by atosiban or placebo [19]. This study was responsible for the FDA nonapproval of atosiban in the USA. However, in Europe, many studies were carried out and did not confirm it. Atosiban is widely used in clinical practice because of its low side effects profile [5, 6]. A german meta-analysis based on 6 randomised trials, among them 3 double blind studies, confirmed a similar tocolytic action for atosiban and *β* adrenergic receptor agonists. A significantly low incidence of adverse effects is reported. Moreover, a lower cost saving in terms of hospital length and extra tests for excluding morbidity causes is found for the atosiban treated patients when compared to continuous fenoterol administration controls [12]. In conclusion, atosiban seems to be an adequate therapeutic choice for effective tocolysis with a low maternal and fetal adverse effects profile.

### 3.6. Calcium-Channel Blockers

These agents are interfering with the calcium ions transfer through the myometrial cell membrane. They decrease intracellular free calcium concentration and induce myometrial relaxation [2–4].

Nifedipine is the most commonly used drug for preterm labour inhibition at a daily dose of 30–60 mg daily. Randomised controlled trials report a similar tocolytic effect for nifedipine compared with *β* adrenergic receptor agonists [20]. Unfortunately, there is no placebo-controlled studies available to confirm it. A Cochrane Database review meta-analysis published in 2003, reported a decreased number of deliveries within 7 days following treatment and also, a reduced incidence of neonatal respiratory distress syndrome [21]. A recent systematic review based on 26 trials and 2179 patients confirms a higher efficiency and a lower side effects incidence in the nifedipine group compared to *β* adrenergic receptor agonists-treated patients [22]. These data confirm that nifedipine is a efficient tocolytic agent, with an easy oral route of administration, few side effects, and a low neonatal complications rate. However, it should be used with caution in patients with compromised cardiovascular condition as they may be at risk of pulmonary oedema and cardiac failure [5].

### 3.7. Progesterone and 17-*α*-Hydroxyprogesterone Caproate

Progesterone is a steroid hormone secreted by the corpus luteum and by the placenta after 8 weeks of gestation. It has a physiological effect on uterine quiescence mediated by a direct effect on intracellular calcium concentration and prostaglandin synthesis [1, 2, 5, 9]. Several randomised trials reported a significantly reduced incidence of preterm birth in patients at risk treated either with weekly intramuscular 17-*α*-hydroxy progesterone caproate [23] or daily vaginal micronized progesterone [24, 25] from 24 to 34 weeks. But these treatments showed no benefit in terms of perinatal mortality and morbidity [2, 5, 23–25]. The vaginal route of progesterone administration is associated with less side effects such as sleepiness and headaches [4, 5]. Although these treatments seem effective in patients with previous history of preterm birth or with a short cervix, it is essential to collect more data in large randomised controlled trials for confirming its potential benefit in the prevention of preterm delivery.

### 3.8. Antibiotics

Infection is one of causal factors of preterm labour with an incidence of 20–40%, especially before 30 weeks [1, 2]. Antibiotics use for preventing preterm labour has been largely studied [5, 28–30]. In the presence of preterm labour with intact membranes, the prophylactic administration of antibiotics is not recommended as there is little evidence of benefits [28]. But if there is a preterm rupture of the membranes (PROM), a meta-analysis based on 22 studies including more than 6000 patients, shows a significant decrease of preterm delivery and chorioamnionitis rate in the treated group [29]. Neonatal complications were also lower in this population [4, 29]. In bacterial vaginosis associated with pregnancy, antibiotics were found to eradicate infection but they showed no effect on the incidence of preterm delivery [30]. In conclusion, PROM is the only clinically proved indication for using antibiotics in order to prevent preterm birth [29].

## 4. Discussion

There are many possible interventions aiming to treat this multifactorial syndrome called preterm delivery. As described here, only some drugs have been proved to be effective on the contraction process, but there are no clear evidence of associated improved neonatal outcome. Some drugs are used as first-line single therapy such as *β* adrenergic receptor agonists and atosiban in Europe [11, 12]. In severe cases, combined therapy could be offered but should be restricted because of adverse effects addition. A Dutch prospective study based on 1920 women, reported that the overall incidence of severe adverse effect is doubled when a multiple-drug regimen is chosen [27]. The literature review evidences that there are still insufficient data regarding some therapies such as the effectiveness of progesterone in the absence of previous medical history and the role of antibiotics, bed rest, and maintenance therapy [5, 31].

Specific conditions are subject to discussion: in multiple pregnancies, expanded blood volume and anaemia may predispose to pulmonary oedema when tocolytic agents such as *β* adrenergic receptor agonists, magnesium sulphate, and calcium channel blockers are prescribed. In these pregnancies, atosiban, with its low side effects incidence, seems to be the safest choice.

The role of tocolysis in PROM allows pregnancy prolongation for corticosteroids administration but has not been reported to significantly improve neonatal outcome [32]. Is long-term therapy effective? There is no clinical evidence on published trials and systematic review to justify tocolytic therapy maintenance except for atosiban [31].

A critical review about tocolysis points to the potential risk of delaying preterm delivery specially in case of infectious or inflammatory process and does not evidence an improved neonatal outcome as tocolysis is often associated with corticosteroids administration [26].

## 5. Conclusions

Prevalence of preterm birth has increased during the last decades and it is a real public health concern. Management with tocolytic drugs aims to stop uterine contractions and to prevent neonatal risks associated with prematurity by in utero transfer of the pregnant patient in a tertiary specialized centre and by corticosteroids administration [1, 2, 7]. 

Our review of several studies and meta-analyses reported on Table 1 confirm the efficacy of *β* adrenergic receptor agonists, prostaglandin-synthetase inhibitors, and atosiban for delaying delivery for 24–48 hours [2, 5, 6, 10, 11, 17]. 

In terms of maternal and fetal safety, the overall prevalence of severe side effects associated with tocolysis is around 1% and is more frequent in multiple therapies, multiple gestation, and preterm rupture of the membranes [27]. Atosiban is our first choice drug for safety, followed by prostaglandin-synthase inhibitors and nifedipine [2, 5, 6, 12, 27]. 

For the future, tocolytic drugs development should aim to reach a better efficacy in terms of pregnancy prolongation and a lower adverse effects profile. A better understanding of the regulation of myometrial contractility and the detection of specific maternal or fetal parameters should be used for new tocolytic strategies. Last generation of oxytocin receptor antagonists such as barusiban could be more efficient and have less affinity for the vasopressin receptors [9]. Specific COX-2 inhibitors or “coxibs,” prostaglandin receptors antagonists could be promising tocolytic alternatives [2, 4, 9, 18].

## Figures and Tables

**Table 1:** Effects of currently used tocolytic drugs.

Drugs	Effects	Outcome	Side effects	Studies
*β* AdRA	Decrease cAMP	Delay D 2–7 days	Cardiovascular	RCT [5, 11, 12]
			Metabolic	Meta-analysis [2, 10]

NO donor	Increase cGMP	Delay D 2 days	Cardiovascular	Small series [2, 5]

MgSO_4_	Decrease IC Ca++	No tocolytic effect	Neurological	RCT, meta-analysis
			Metabolic	[2, 4–6, 13, 14]
			Perinatal mortality	
		Fetal neuroprotection		RCT [15, 16]

PgSI	On gap junction	Delay D 2–7 days	Gastrointestinal	RCT, meta-analysis
	Decrease IC Ca++		Fetal kidney function	[2, 4–6, 17, 18, 26]
			Premature closure ductus arteriosus	

Ox RA	Competition with receptor binding	Controversial efficiency	IUGR? Mortality?	Review [2]
			Few side effects	RCT, meta-analysis
				[4, 5, 9, 11, 12, 19, 27]

Ca++ CB	Decrease IC Ca++	Delay D 7 days	Cardiovascular	No placebo RCT
		Decreased neonatal morbidity		Comparative RCT
				[2, 4, 9, 20–22]

Progesterone	Reduction preterm delivery in high-risk patients			RCT [23, 25]
	Decrease IC Ca++		Sedative	
	Decrease Pg synthesis		Liver cytolysis	[24, 26]

## Abbreviations

*β* AdRA: *β* adrenergic receptor agonist
MgSO_4_: Magnesium sulphate
PgSI: Prostaglandin synthase inhibitor
Ox RA: Oxytocin receptor agonist
Ca++ CB: Calcium channel blocker
Delay D: Delay for delivery
RCT: Randomized controlled trial
DA: Ductus arteriosus
IC Ca++: Intracellular calcium concentration
IUGR: Intrauterine growth retardation.
